# First person – Emilie Auxerre-Plantié and Tanja Nielsen

**DOI:** 10.1242/dmm.047902

**Published:** 2020-12-21

## Abstract

First Person is a series of interviews with the first authors of a selection of papers published in Disease Models & Mechanisms, helping early-career researchers promote themselves alongside their papers. Emilie Auxerre-Plantié and Tanja Nielsen are co-first authors on ‘Identification of *MYOM2* as a candidate gene in hypertrophic cardiomyopathy and Tetralogy of Fallot, and its functional evaluation in the *Drosophila* heart’, published in DMM. Emilie conducted the research described in this article while a postdoctoral researcher in Prof. Silke Rickert-Sperling's lab at Charité–Universitätsmedizin Berlin, Germany. She is now a postdoctoral researcher in the lab of Dr Lucas Waltzer at the Gred Institute, Clermont-Ferrand, France, investigating (epi)genetics in *Drosophila* during muscle and heart development. Tanja conducted the research described in this article while a PhD student in Prof. Silke Rickert-Sperling's lab. She is now a PhD student in the lab of Prof. Rolf Bodmer at the Sanford Burnham Prebys Medical Discovery Institute, La Jolla, CA, USA, investigating the genetic basis of congenital heart diseases using *Drosophila* as a genetic heart model.


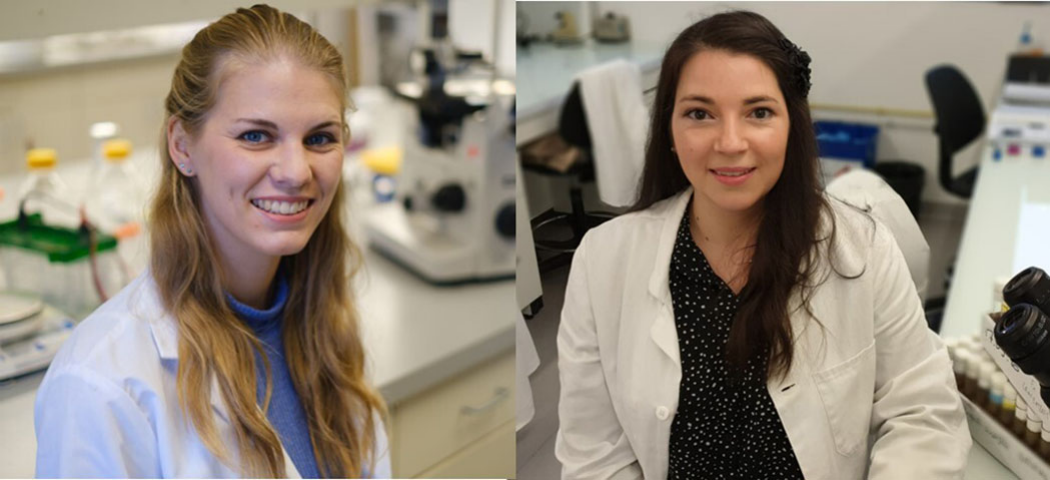


**Emilie Auxerre-Plantié and Tanja Nielsen**

**How would you explain the main findings of your paper to non-scientific family and friends?**

**EAP/TN:** Every 30 seconds, a baby is born with congenital heart disease (CHD) and, therefore, CHD is one of the leading causes of death worldwide, making it a research priority to understand its causes. In the past two decades, researchers have been able to access more and more patient genomic data due to next-generation sequencing. With this technology, thousands of mutations in CHD patients have been identified and have created a great need to test these in animal models. Therefore, we need simple animal models like the fruit fly, *Drosophila*, which has a heart with many remarkably similar features to the human heart. Our results suggest that MYOM2 plays a critical role in maintaining robust heart function, as flies mutant for MnM (the MYOM2 counterpart in the fly) develop cardiac defects. Moreover, MYOM2 is a candidate gene for several types of heart diseases, such as hypertrophic cardiomyopathy and the severe CHD, Tetralogy of Fallot, as MYOM2 was found to be mutated in these CHD patients.

“Every 30 seconds, a baby is born with congenital heart disease […]”

**What are the potential implications of these results for your field of research?**

**EAP/TN:** As only 25% of all CHDs can be explained, in part by gene mutations, we aim to identify novel candidate genes involved in CHD with our approach using *Drosophila* as a genetic heart model, as demonstrated here for *MYOM2*. By studying patient-derived genetic variants and their effect on heart development and function, we hope to eventually use this knowledge to uncover novel genetic markers/risk factors for prenatal diagnostic or long-term predictions for heart failure. Over the years, *Drosophila* has become an established genetic heart model. With this single gene approach, we want to provide an example of how *Drosophila*, with its benefits of rapid generation time and a large amount of available genetic tools, can be used in the future to study complex human diseases, including CHDs.

**What are the main advantages and drawbacks of the model system you have used as it relates to the disease you are investigating?**

**EAP/TN:** As mentioned before, the fruit fly is a very easy-to-handle animal model and develops quickly as we obtain the next generation of specimens in 10 days. Therefore, we can conduct studies more easily, faster and cheaper compared to mammalian models. Moreover, we have access to numerous genetic tools available with *Drosophila*.

About 80% of human disease genes have a fly counterpart and as the heart is dispensable for the early survival of the fly, we are able to study severe mutations in adult flies that would be embryonic lethal in other model organisms. Although heart morphology in flies is different from mammals, as it is a linear tube compared to the four-chambered human heart, the pathways and genes that control cardiac development and function are very well conserved between both species. Altogether, this makes *Drosophila* a model of choice for testing the impact of mutations on cardiac development and physiology.

“About 80% of human disease genes have a fly counterpart […]”

**What has surprised you the most while conducting your research?**

**EAP:** As this research project was a collaboration between *non-Drosophila* and *Drosophila* researchers, the discussions were always very interesting and intense, and made me learn a lot. Moreover, as I got my PhD in France and did this postdoc in Berlin, it was an opportunity to discover another way of working, as it was a bit more structured, and helped me to improve my English. I was enthusiastic about applying the knowledge I acquired during my PhD (*Drosophila* as a cardiac model) to better understand human diseases, in particular CHDs.

**Figure:**
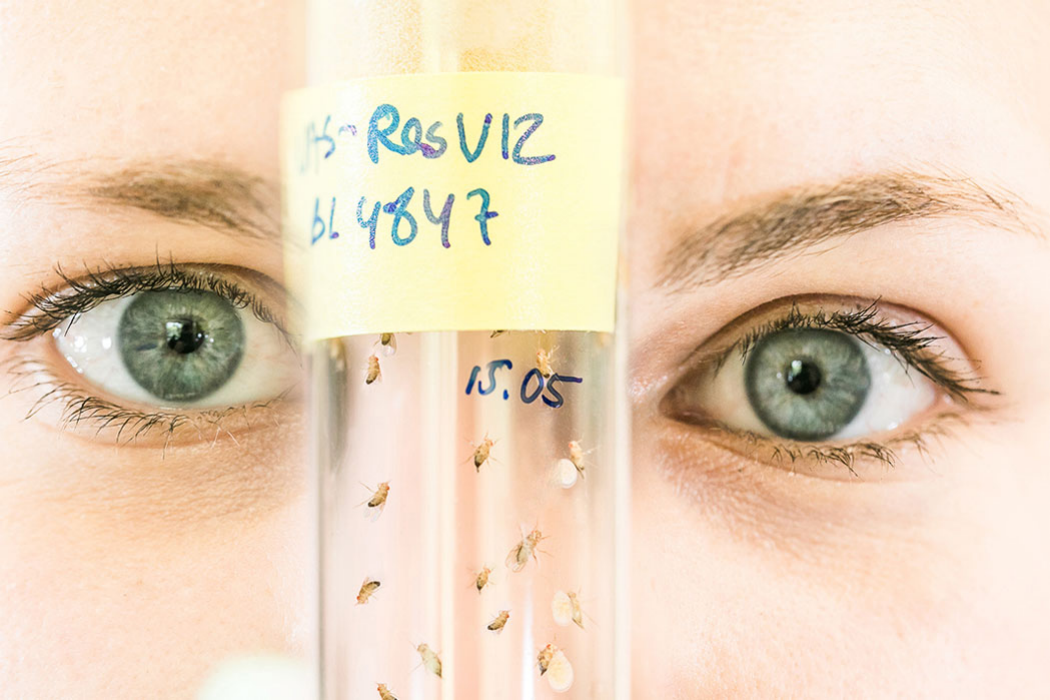
**The fruit fly through the eyes of a Drosophilist – a powerful genetic pioneer organism to study congenital heart diseases!**

**TN:** Starting my PhD, I was most surprised when getting the first view into, what one could call, the ‘*Drosophila* research world’! For example, in addition to very detailed and rich databases like ‘Flybase’, there are fly stock centres around the world that harbour huge collections of fly stocks that can be used as genetic tools to study the genetics and molecular mechanisms in the fly. Furthermore, I was now part of a very well-connected and collaborative *Drosophila* community. *Drosophila* is a very well-studied model organism, and as a newbie, it was impressive to realize that the work of many Drosophilists before me, was actually the foundation to explain many of today's established principles across organisms, i.e. in organogenesis and gene regulation. For example, one of the main cardiac transcription factors, Nkx2-5, was first identified in the fly by Rolf Bodmer in 1993.

**Describe what you think is the most significant challenge impacting your research at this time and how will this be addressed over the next 10 years?**

**EAP/TN:** The main challenge is to apply our knowledge to conduct translational (from bench to bedside) research and improve the health of CHD patients. Owing to the complexity and heterogeneity of CHD, one of the biggest obstacles of the field is to understand and solve the patient-specific genotype-phenotype relationship. While over the years an increasing number of genes implicated with CHD were identified via linkage analysis or genome-wide association studies, the majority of cases still remain unexplained.

For the future, the genetic heterogeneity of many diseases will necessitate genomic and functional analysis of potential CHD-associated variants on a large scale in order to identify novel risk factors/modifiers, and to understand how these regulate heart development. Finally, we hope to be able to identify combinations of detrimental mutations (with underlying complex genetics) responsible for most CHDs, to offer personalized medicine.

**What changes do you think could improve the professional lives of early-career scientists?**

**EAP:** As I got my PhD in 2016 in Clermont-Ferrand (France) and am currently in my second postdoc, I think retrospectively that mentoring was not properly established in our university. Currently, there is a new strategy for helping graduate students to make the right choices for their future career (postdoc/next lab/perspectives). Moreover, I think that in academic research, the concept of networking should be encouraged, as in pharmaceutical/private research to help students have a better career plan.

Finally, I think there is a great lack of financial support, maybe more specifically in France, as was raised recently by Emmanuelle Charpentier, the 2020 Nobel Prize winner for Chemistry for the gene-editing CRISPR/Cas9 technology. There are early-career scientists motivated intellectually to pursue a career in science but they need the appropriate financial support to achieve their goals.

**TN:** From my perspective as a fourth year PhD student and based on my experiences so far, I think that the structured PhD programs nowadays are very well-organized and do a good job of supporting early scientists, and educate and prepare graduate students for a scientific career. They target not only the development and improvement of scientific-related skills but also soft skills, like leadership or presentation skills. I greatly benefited from various courses, not just those offered by the PhD programs I was enrolled in. Of course, mentorship plays a big role in professional development, especially in earlier stages. In addition to the lab head providing main scientific guidance and mentorship, I think it would be beneficial if every PhD student had access to mentoring programs, including more experienced postdocs, experts from industry, or even people outside the field, to become acquainted with additional career perspectives and to discuss career goals and directions.

**What's next for you?**

**EAP:** I am currently working on an exciting project, focused on epigenetics and muscle development and function. I will finish my second postdoc in a year and hopefully publish. Then, I would love to apply for a permanent research position in France but, as in other countries, it is becoming more and more difficult to succeed, so I do not exclude working in industry or switching to teaching.

**TN:** For my future career, one aspect that is especially attractive to me regarding my current project, and highly motivates me, is the translational aspect. It is exciting to work together with the physicians, which can give direct insights from the patient's side and to connect them to my experiments. This is exactly what I consider as my future career: to be at the interface between cardiac research and heart disease patients by aiming for a job in clinical cardiology studies. After receiving my PhD, I plan to first gain further experience in the heart field (preferably using mammalian models) and expand my knowledge in academia as a postdoc, and eventually transition to participating in clinical studies.
